# Report of the Joint Meeting of the 6th Asian Congress on Environmental Mutagens and the 48th Annual Meeting of the Japanese Environmental Mutagen Society, Tokyo, November 18–20, 2019

**DOI:** 10.1186/s41021-020-00170-2

**Published:** 2020-11-24

**Authors:** Masamitsu Honma

**Affiliations:** National Institute of Health Sciences, 3-25-26 Tonomachi, Kawasaki City, Kanagawa 210-9501 Japan

**Keywords:** Asian Association of Environmental Mutagen Societies (AAEMS), Asian Congress on Environmental Mutagens (ACEM), Japanese Environmental Mutagen Society (JEMS), Tokyo

## Abstract

The 6th Asian Congress on Environmental Mutagens (ACEM) was held at Hitotsubashi Hall, Chiyoda City, Tokyo on November 18–20, 2019, in conjunction with the 48th Annual Meeting of the Japanese Environmental Mutagen Society (JEMS). Ninety international delegates from Australia, China, Czechia, France, Germany, India, Iran, Italy, Korea, the Netherlands, the Philippines, the UK, and the USA, along with 340 Japanese delegates and students, participated. During the conference, one keynote lecture, seven symposia, and one workshop were held under the theme of “Innovations towards Environmental Mutagen and Genome Research Originating from Asia.” In the general presentation, 34 oral presentations and 138 poster presentations were made, accompanied by lively discussions. The organizers would like to express their sincere gratitude to those who attended the conference and made it a great success.

## Opening remarks

**Fig. 1:**
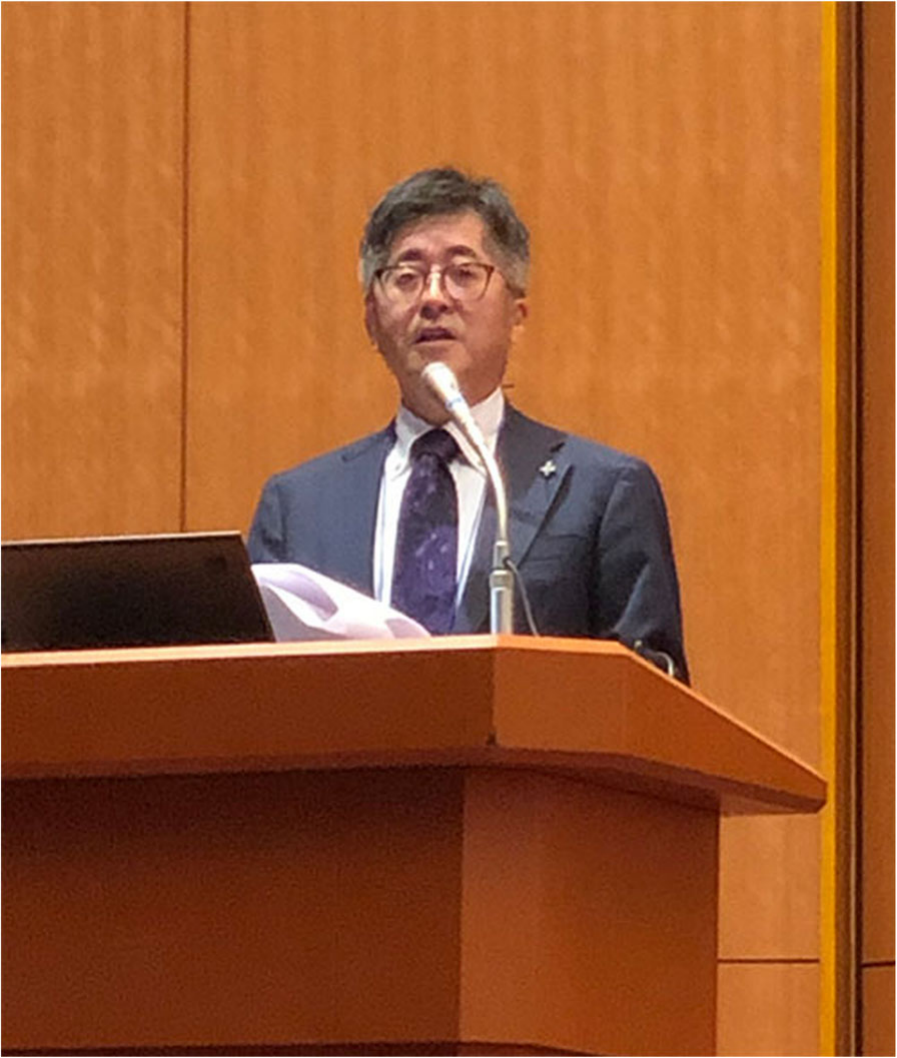
Opening remarks by Dr. Honma

The Joint Meeting of the 6th Asian Congress on Environmental Mutagens (ACEM) and the 48th Annual Meeting of the Japanese Environmental Mutagen Society (JEMS) was held at Hitotsubashi Hall, Chiyoda City, Tokyo on November 18–20, 2019. Opening remarks were given by Dr. Honma, Organizing Committee Chair of ACEM/JEMS 2019 (Fig. 1), in which he discussed health and environmental issues in Asian countries, the role and history of the Asian Association of Environmental Mutagen Societies (AAEMS), and the theme of the conference.

The Asia-Pacific region is the largest in the world, and exhibits distinctive lifestyles that reflect a combination of different cultures, customs, and economies. Consequently, our region faces a variety of unique environmental and health issues. In particular, exposure to mutagens in the living environment, work settings, medical care, and ordinary diet causes serious health problems including cancer and other diseases. AAEMS was established in 2004 to encourage research on environmental mutagenesis and to address these problems. There are currently eight member countries of AAEMS: China, Korea, the Philippines, Thailand, India, Iran, Australia, and Japan. The first meeting of AAEMS was held in Japan (Kitakyushu) in 2007, followed by Thailand (Pattaya), China (Hangzhou), India (Kolkata), and South Korea (Incheon), with the 6th ACEM being held again in Japan. The theme of this conference was “Innovations towards Environmental Mutagen and Genome Research Originating from Asia.”

## Symposia A: environmental mutagen issues in Asian countries

Asian countries, which have large populations, varying levels of development, and diverse cultures and lifestyles, have different environmental and health problems from those in Europe and the United States. In Japan, the 2011 Fukushima Daiichi Nuclear Power Plant accident brought global attention to the serious problems of radiation, while residents suffered from rumors connected to radiation. The following four symposia were held to better understand and share health and environmental problems caused by environmental mutagens in Asian countries, and to stimulate future research.

- What is the problem now in environmental mutagen research in Asia?
- Genotoxicity of Aristolochic acid and other plant toxicants in Asia
- Exposure and health risk of air pollutants in Asia
- Risk assessment of low-dose irradiation: current situation in Fukushima and other Asian cities

**Fig. 2:**
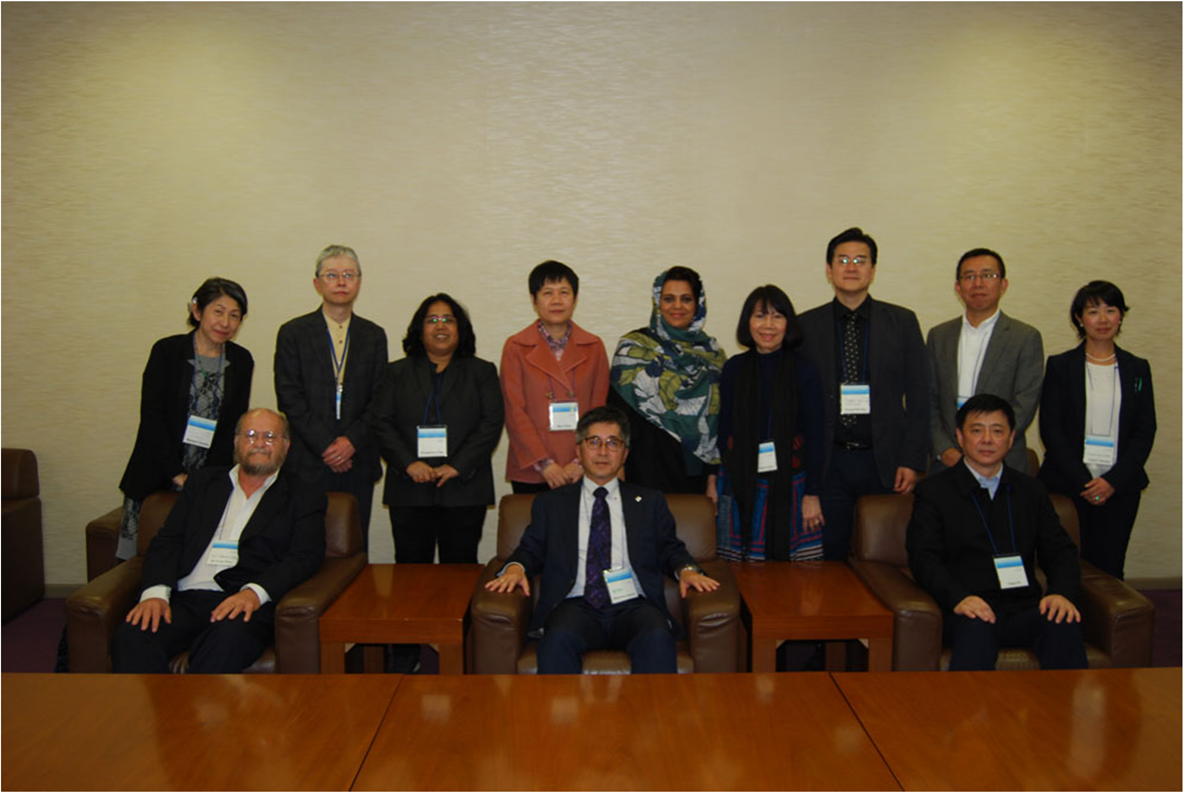
AAEMS executive committee members meeting

In particular, in “What is the problem now in environmental mutagen research in Asia?” representatives from all eight member countries were present at ACEM for the first time (Fig. 2). They introduced the state of their own research on environmental mutagens and the activities of their EMS societies (representatives from the Philippines were absent due to sudden illness).

## Symposia B: genome research in Japan and Asian countries

At the JEMS business meeting, it was decided to change the society’s name from “The Japanese Environmental Mutagen Society” to “The Japanese Environmental Mutagen and Genome Society.” The word “genome” is gaining prominence in life science research, and in recognition of this we held the following two symposia and one special Japanese session.

- Unravelling of cancer in Asia using genomic and adductomics approaches
- Molecular mechanisms of genome mutation
- Where should JEMS go? (in Japanese)

The membership of JEMS has been declining and currently stands at about 500. This is a crisis for JEMS’ survival. In the Japanese session, JEMS members of various ages and backgrounds, including academia, industry, Contract Research Organizations (CROs), and regulatory agencies, met and discussed the future of the society. Are there any serious environmental mutagens in Japan today that are potentially carcinogenic in humans? Also, even if one exists, can it be elucidated in the research areas targeted by JEMS? This is a common question among researchers in EMS societies around the world, and highlights the need to abandon the past and to pioneer new research areas.

## Keynote lecture and workshop: in silico genotoxicity assessment

\While Asian countries have serious health issues due to environmental pollution, Information Technology computational research is an active area of study. In the field of genetic toxicology, an International Council for Harmonization of Pharmaceutical Regulations (ICH) guideline approved quantitative structure-activity relationship (QSAR) approaches to evaluate the mutagenicity of impurities as an alternative to the Ames test. This guideline has triggered growing interest in QSAR [1]. Dr. Romualdo Benigni from Italy thus delivered a keynote lecture on the subject, and a subsequent workshop was held.

- QSAR is an essential tool of integrative assessment strategies (Keynote)
- ICH-M7 QSAR/expert judgment workshop - iGenotox Challenge prediction

Dr. Benigni is a pioneer of QSAR research in the field of genetic toxicology, and his talk comprehensively described the QSAR approach from basics to applications [2]. The workshop was divided into two sessions. The first concerned the verification of the ability of QSAR tools to predict Ames mutagenicity, and case studies on the evaluation of chemical mutagenicity through a combination of QSAR and expert judgment. Eight researchers from academia, regulatory agencies, consultants, companies, and QSAR vendors spoke. During the second session, three pharmaceutical industry researchers addressed the management of mutagenic impurities based on chemical structure. This workshop provided simultaneous interpretation in English and Japanese and was very well received by attendees unfamiliar with QSAR who were able to enhance their understanding on this topic. This was a unique workshop that has not been used by other EMS societies, and one which will be important for JEMS to continue.

## General session: platform session and poster session

**Fig. 3:**
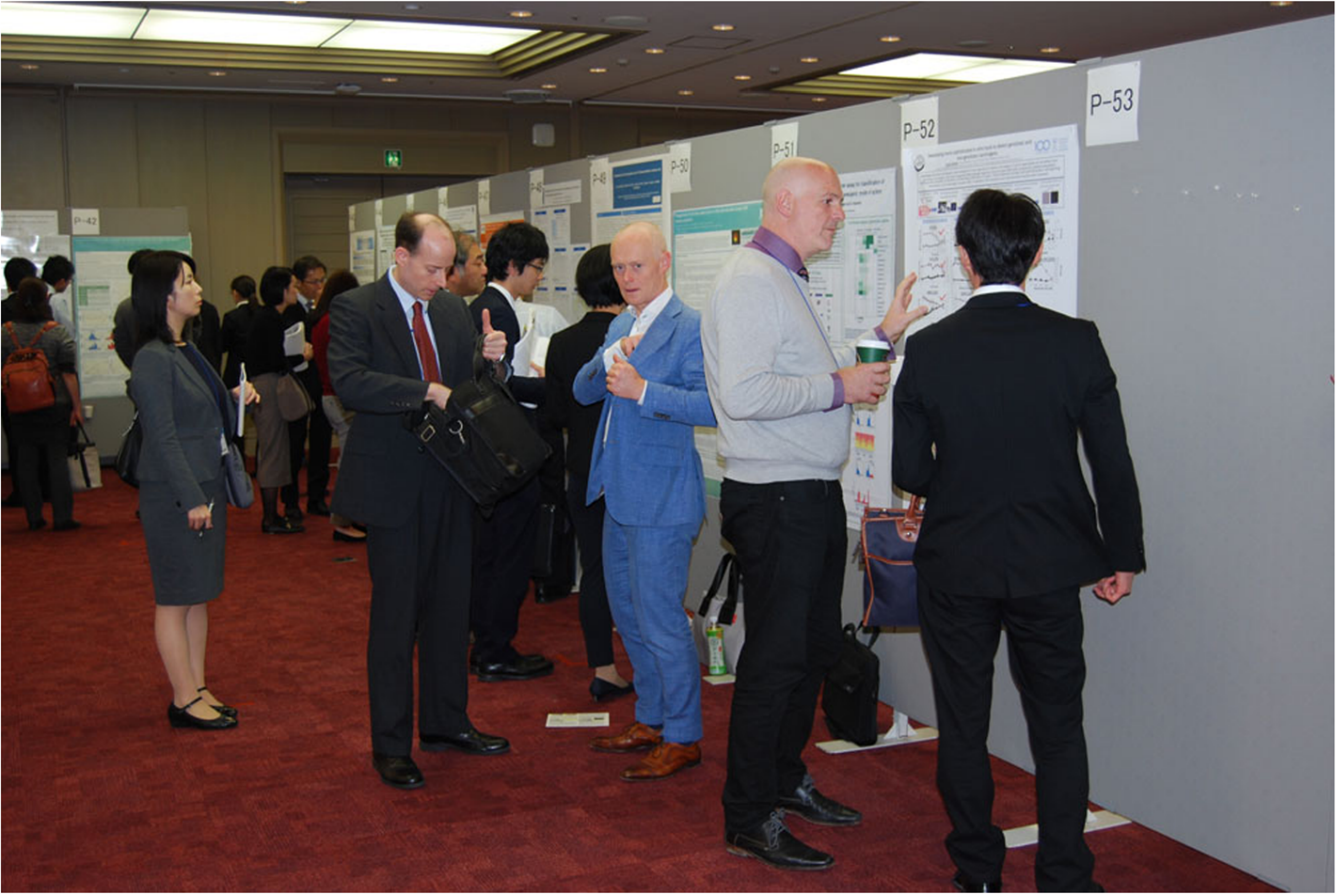
Poster session

**Fig. 4:**
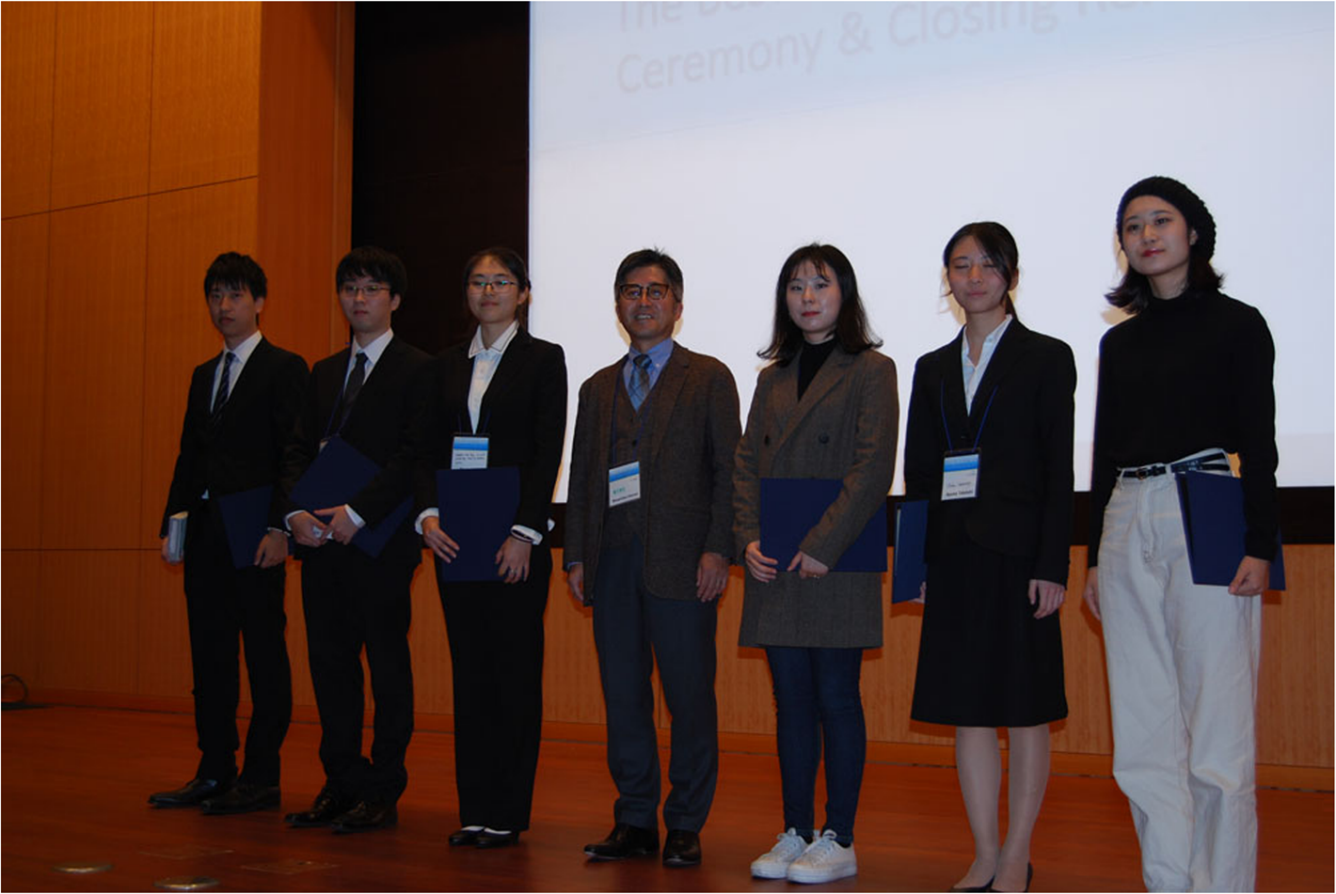
Best presentation award winners

ACEM/JEMS 2019 hosted six platform sessions and two poster sessions. In total, 34 oral presentations and 138 poster presentations were made (Fig. 3). Six presentations were selected as the best of the general session, with the following students or early-career researchers being awarded: Ai Ueshima (Osaka City University, Japan), Hyun Soo Kim (Dongguk University, Korea), Ayuna Takeishi (Chiba University, Japan), Xinyue You (Shanghai Jiao Tong University, China), Toshihide Takeshita (Yokohama City University, Japan), and Shota Ueda (Fukuoka University, Japan) (Fig. 4).

## Social program

**Fig. 5:**
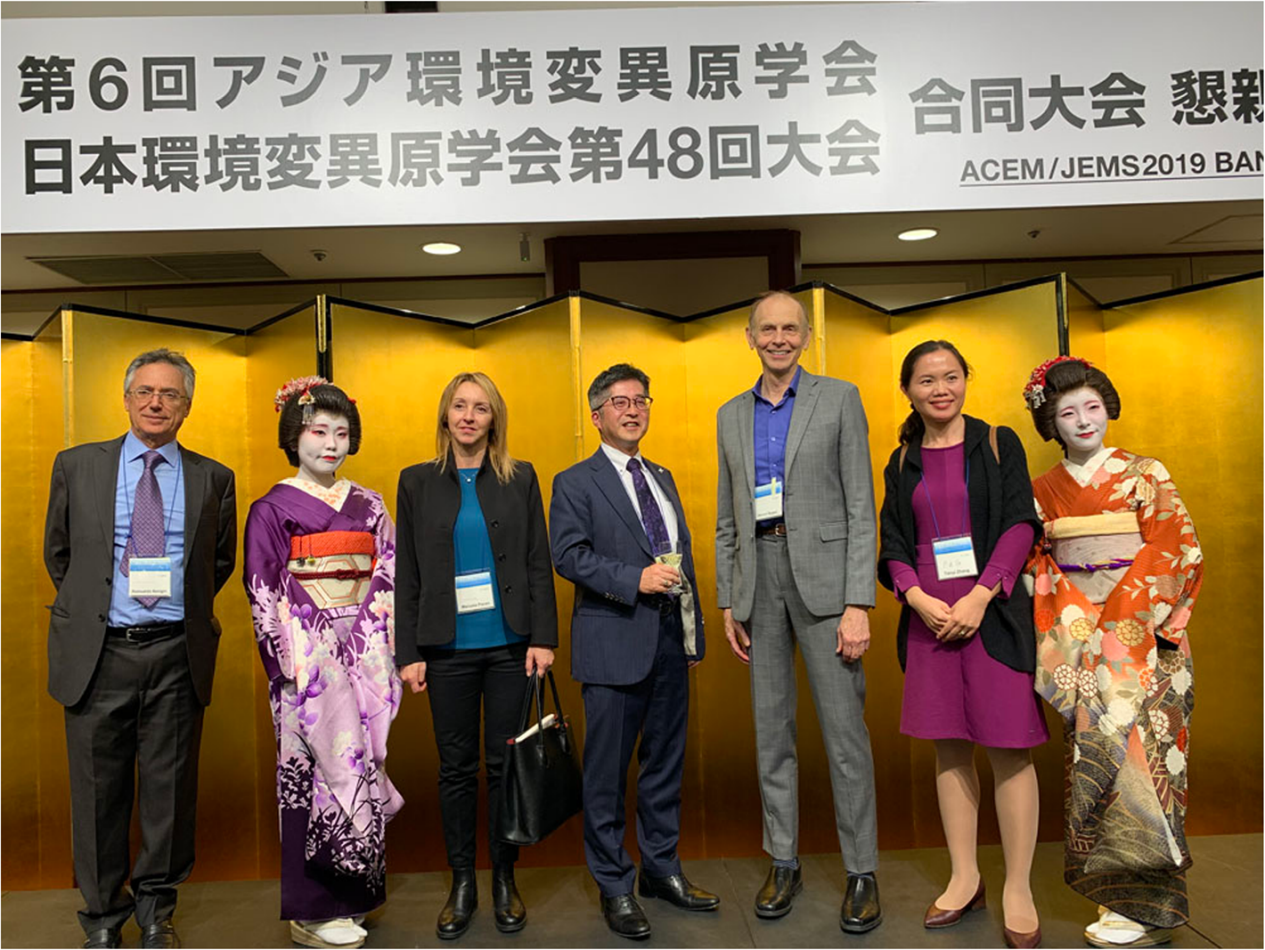
ACEM/JEMS 2019 banquet

A social gathering was held at Josui Hall on the evening of the first day. We wanted participants from overseas to experience a Japanese atmosphere, so we invited “Kimono-Ladies” to perform Japanese dance and singing. The participants also took photos together as they wished, and left with good memories (Fig. 5).

## Conclusion

ACEM/JEMS 2019 was a great success with 430 participants from 18 countries. The organizers would like to thank the scientists, EMS representatives from Asian countries, and students for attending the conference and contributing to its success. Dr. Homma also thanks colleagues from JEMS, MMS, and NIHS, and the staff of Sendai Kyodo Insatu Co., Ltd., all of whom helped to prepare the conference and offered unstinting encouragement. Dr. Honma retired as president of AAEMS at the end of 2019, with Dr. Jia Cao (Third Military Medical University, China) taking up the post from 2020. The 7th ACEM will be held in Qingdao, China in 2022, and will be hosted by Dr. Yuxin Zheng (Qingdao University, China). We hope that AAEMS will develop further as more Asian countries become members, and that the next ACEM will be successful and attract many participants.

## Data Availability

Not applicable.

## Abbreviations

AAEMS: Asian Association of Environmental Mutagen Societies
ACEM: Asian Congress on Environmental Mutagens
JEMS: Japanese Environmental Mutagen Society
CRO: Contract research organization
ICH: International Council for Harmonisation of Technical Requirements for Pharmaceuticals for Human Use
QSAR: Quantitative structure-activity relationship
